# ‘We don’t live in a harm reduction world, we live in a prohibition world’: tensions arising in the design of drug alerts

**DOI:** 10.1186/s12954-022-00716-3

**Published:** 2023-01-09

**Authors:** Isabelle Volpe, Rita Brien, Jasmin Grigg, Stephanie Tzanetis, Sione Crawford, Tom Lyons, Nicole Lee, Ginny McKinnon, Caitlin Hughes, Alan Eade, Monica J. Barratt

**Affiliations:** 1 Social and Global Studies Centre, RMIT University, Melbourne, Australia; 2 Drug Policy Modelling Program, Social Policy Research Centre, UNSW Sydney, Sydney, Australia; 3 Turning Point, Eastern Health Statewide Services, Richmond, Australia; 4 Monash Addiction Research Centre, Eastern Health Clinical School, Monash University, Melbourne, Australia; 5 CanTEST (Direction Health Services, Pill Testing Australia, Canberra Alliance for Harm Minimisation and Advocacy), Canberra, Australia; 6 Department of Health, Victoria State Government, Melbourne, Australia; 7 360Edge, Melbourne, Australia; 8 National Drug Research Institute, Curtin University, Perth, Australia; 9 Law and Commerce, Flinders University, Adelaide, Australia; 10 National Drug and Alcohol Research Centre, UNSW, Sydney, Australia; 11 Safer Care Victoria, Melbourne, Australia; 12 Department of Paramedicine, Monash University, Melbourne, Australia; 13 Digital Ethnography Research Centre, RMIT University, Melbourne, Australia

**Keywords:** Drug alerts, Early warning system, Workforce, Drug risk communication, Co-design, Drug checking, Credibility, Stigma, Harm reduction, Tensions

## Abstract

**Background:**

Drug alerts designed for health and community workforces have potential to avert acute harms associated with unpredictable illicit drug markets, by preparing workers to respond to unusual drug-related events, and distribute information to service users. However, the design of such alerts is complicated by diverse needs of individuals, and broader socio-political contexts. Here, we discuss the tensions that arose in the process of co-designing drug alert templates with health and community workers.

**Methods:**

We conducted five in-depth digital co-design workshops with 31 workers employed in alcohol and other drug and urgent care settings. Our approach to analysis was informed by Iterative Categorisation and reflexive thematic analysis methods.

**Results:**

We identified five key tensions. First, there is a need to provide comprehensive information to meet the information needs of a diverse group of workers with varying knowledge levels, while also designing alerts to be clear, concise, and relevant to the work of individuals. Second, it is important that alerts do not create ‘information overload’; however, it is also important that information should be available to those who want it. Third, alert design and dissemination must be perceived to be credible, to avoid ‘alert scepticism’; however, credibility is challenging to develop in a broader context of criminalisation, stigmatisation, and sensationalism. Fourth, alerts must be carefully designed to achieve ‘intended effects’ and avoid unintended effects, while acknowledging that it is impossible to control all potential effects. Finally, while alerts may be intended for an audience of health and community workers, people who use drugs are the end-users and must be kept front of mind in the design process.

**Conclusions:**

The co-design process revealed complexities in designing drug alerts, particularly in the context of stigmatised illicit drug use, workforce diversity, and dissemination strategies. This study has highlighted the value of developing these important risk communication tools with their target audiences to ensure that they are relevant, useful, and impactful. The findings have informed the development of our drug alert prototypes and provide local context to complement existing best-practice risk-communications literature.

**Supplementary Information:**

The online version contains supplementary material available at 10.1186/s12954-022-00716-3.

## Background

Illicit drug markets are unpredictable with new substances emerging sporadically [1] and established drugs varying in potency and quality (e.g. through contamination or adulteration) [2]. Consequently, consumers are unable to know with certainty the contents of their drugs, leading to risk of significant harm. In recent years, high strength and contaminated substances have contributed to experiences of acute harm resulting in numerous hospitalisations and fatalities in Australia and globally [3–7]. This has led to multiple calls for drug checking services and early warning systems, in which information about the content of substances would be made available to the public (e.g. [8, 9]). Drug alerts can provide timely information about market changes, such as substances in circulation that are known to be contaminated, adulterated, missold, or otherwise misrepresented), and can inform and encourage harm reduction behaviour [10, 11] with potential to avert acute harms.

As mechanisms for monitoring drug markets are increasingly utilised (e.g. [12–15]), there has been a corresponding increase in research that explores the design and evaluation of these surveillance tools (e.g. [10]). To date, this body of work has focused on drug alerts designed to reduce harm through direct education and behaviour change at the consumer level [11, 16, 17]. Drug alerts designed for relevant workforces as the primary target audience, such as alcohol and other drug (AOD) (e.g. people working directly with people who use drugs in AOD treatment, harm reduction, peer support, or outreach services) and urgent care workers (e.g. hospital emergency department workers, paramedics, nurses and event-based first responders), have received comparatively less attention. Although health and community workers are a crucial point of contact for drug-related issues, information and advice, workforce research has demonstrated that there is little support and education available for these workers discuss novel psychoactive substances and current drug markets [18–23]. As such, drug alerts that target health and community workers are an important opportunity to facilitate broader dissemination of alerts, provide up-to-date information to increase drug literacy, and prepare services to respond to drug market fluctuations or unusual drug-related events [14, 24].

Previous research has explored complexities and tensions in illicit drug risk communication messaging [11, 16, 25]. This research demonstrates the importance of considering social contexts of local ‘drug scenes’ or drug use environments, as well as the characteristics and drug consumption practices of individuals [26]. For example, alerts designed to warn potential consumers of higher-strength substances can be interpreted as conveying information about ‘quality’ (that is, they may be perceived as desirable) rather than ‘risk’ (that is, unknowingly ingesting a drug of a higher strength than usual can increase the risk of overdose) [11, 16, 25]. Similarly, health and community workers that work with people who use drugs have diverse backgrounds (e.g. varying levels of education, personal experiences of and around drug consumption) and work in diverse workplace environments (e.g. clinical, outreach and social service settings). Understanding the nuanced ways messaging will be perceived and interpreted by target audiences can facilitate the design, implementation and effectiveness of drug alerts [27].

In a pilot study (Rapid Drug Alerts for Victoria; RapidDAV), we co-designed a set of drug alert templates with health and community workers (working in AOD and urgent care settings in Victoria, Australia), to optimise their utility and impact in clinical settings. The co-design process and template itself are discussed in a separate article [28]. The project was borne out of an opportunity to utilise drug analysis information from existing data sources to alert the health workforce about any high-risk drugs in circulation. In engaging in the co-design process with health and community workers, we encountered several challenges to designing drug alerts, which we identified to be ‘tensions’ warranting further consideration. In the present study, we aim to explore these tensions arising during the process of co-designing drug alert templates for a clinical audience and discuss avenues of navigating these tensions.

## Methods

### Design

We used an iterative participatory co-design approach to inform the development of the drug alert prototypes (see Methods in [28]). Participatory co-design emphasises the participation of end-users at each stage of the design process, from identifying the needs of participants through to evaluating the implementation of the final products [29]. As such, we conducted workshops with members of the intended drug alert audience of health and community workers. Workshops were broadly guided by a schedule designed to answer the research aims.

### Recruitment and sampling

We recruited from a list of participants who responded to an initial scoping survey for our broader project and consented to be contacted for workshops. More information about sampling methods for the broader project can be found in Brien et al. [28]. All participants who expressed interest in participating in a workshop were invited to attend.

In this study, we use the term ‘health and community workers’ to describe any people working to provide health-related support to people who use drugs. We specifically recruited AOD workers (e.g. peer workers, psychologists, counsellors, and specialists) and urgent care workers (e.g. hospital emergency department workers, paramedics, nurses, and event-based first responders). Participants were eligible if they (i) were either an AOD worker or urgent care worker; and (ii) were either a ‘practitioner’ (i.e. client-facing roles, responsible for the direct provision of drug-related treatment, advice and/or support) or a ‘manager’ (working in managerial roles and responsible for allocating resources or staffing); and (iii) worked in Victoria, Australia.

### Participant characteristics

Participants were 31 people working in AOD (*n* = 24, 77%), urgent care (*n* = 2, 7%), or both (*n* = 5, 16%) settings across Victoria, Australia (metropolitan areas *n* = 22, 71%; non-metropolitan areas *n* = 9, 29%). Of participants, *n* = 20 (65%) identified as practitioners, *n* = 9 (29%) identified as managers working in managerial roles and responsible for allocating resources or staffing, and *n* = 2 (6%) identified as both (Table 1).

**Table 1:** Characteristics of workshop participants

Participant characteristics	Count *N* (%)
Total *N*	31 (100)
*Workplace location*	
Metropolitan	22 (71)
Non-metropolitan	9 (29)
*Workplace setting*	
Alcohol and other drugs ONLY	24 (77)
Urgent care ONLY	2 (7)
Alcohol and other drugs AND urgent care	5 (16)
*Role*	
Practitioner ONLY	20 (65)
Manager ONLY	9 (29)
Practitioner AND manager	2 (6)

### Data collection

Data for this study were collected via five 90 minute in-depth workshops. Four initial workshops were held in December 2020 to generate initial co-design data. One additional workshop was held in September 2021, in which participants from all four initial workshops were invited to participate and discuss the tensions that had been identified in the initial workshops. Workshops were conducted via videoconference, and included interactive activities using a collaborative *Google Slides* (www.google.com/slides/about/) document (e.g. a ‘brainwriting’ activity involved participants writing quick thoughts on digital ‘sticky notes’ on a shared slide), and utilisation of videoconference chat functions. Participants were invited to share their views on the interactive slides, or use the chat function. Participants’ views were then used to prompt discussion. Each workshop was led by two researcher-facilitators (IV and RB), with assistance from one other researcher (MB or JG) who took notes, monitored the chat function, and occasionally asked clarifying questions.

### Data analysis

Three forms of data were analysed: workshop audio recordings, written ‘chat’ data, and screen captures of interactives slides. Audio recordings of workshops were transcribed verbatim using a professional transcription service. Chat data and slide screen captures were exported as PDF files.

All data were imported into NVivo [30] for coding. Our approach to analysis was informed by Iterative Categorisation [31] and reflexive thematic analysis [32–34];we followed Iterative Categorisation procedures from coding through to descriptive analysis, as a method of ‘Familiarisation’ and ‘Coding’ per reflexive thematic analysis [34].

We developed an initial coding frame deductively according to the project research questions and questions from the workshop schedule We then developed the coding frame based on open coding of the first workshop transcript, as well as field notes and observations from the workshops [31]. After IV, RB, MB and JG agreed on this coding frame, IV and RB each coded approximately half of the interviews independently and then reviewed the other’s coding to check for consistency and generate additional insights [see Additional file 1 for the final coding framework).

For the initial descriptive analysis, we exported the coding files to Microsoft Word. We grouped and summarised key ideas and sentiments, reviewing and re-grouping ideas after each code was analysed [32–34]. Finally, we collated all summary files to to analyse connections across different codes.

At the point of descriptive analysis, our methods diverged from those outlined in Brien et al. [28] and continued to follow reflective thematic analysis phases of 'Developing', 'Reviewing', and 'Defining' themes [34]. In the process of developing prototypes based on the descriptive analyses and familiarisation with the data, we identified tensions in relation to the design of drug alerts and alert systems. This prompted us to inductively develop 'tension' themes where participants’ needs and preferences diverged, contradicted each other, or were seemingly incompatible. IV mapped candidate themes and reviewed these in discussions with RB and MB, refining the themes based on interconnections, clarity of organisation, and researcher knowledge of existing literature.

## Results and discussion

We present five interrelated tensions produced in workshops with health and community workers to inform the development of drug alert prototypes:

1. Providing clear, concise, and relevant messaging while designing alerts for a diverse range of audiences and knowledge levels.
2. Minimising alert fatigue while providing as much information as possible.
3. Trust and safety in a prohibition world.
4. Avoiding unintended alert consequences while acknowledging that it is not possible to manage all risks.
5. Designing drug alerts for workers, when consumers are the affected population.

### TENSION 1: Providing clear, concise, and relevant messaging while designing alerts for a range of audiences and knowledge levels

Health and community workers who work with people who use drugs are a vastly heterogeneous group. Participants discussed that this diversity must be kept front of mind when developing drug alerts, to ensure that the messaging is clear, appropriate, and understandable by the whole target audience. Although we initially anticipated that there may be differing information preferences and needs among health and community workers (who have different educational backgrounds, varying levels of experience, and work in different settings and locations), the diversity of this population’s needs and preferences was strongly emphasised by participants.

Participants described varying knowledge levels and contextual backgrounds in their workforces. It was also evident that participants themselves had different levels of knowledge about drugs, harm reduction, and treatment. For example, at several points during group conversations, participants and facilitators used slang, abbreviations, and technical terminology which was not understood by all participants. This drew attention to mixed knowledge levels that are likely to influence how workers conceptualise, engage with, and respond to the topic of drugs and drug use [35–38].

To maximise utility when developing drug alerts for health and community workers, participants suggested that it is important to think broadly and critically about who might benefit from receiving such alerts.

*Remember that not everyone that’s going to be using this stuff is going to be an AOD clinician. There’s going to be maternal child health nurses, school teachers, a whole lot of other people*. AOD manager, #57

This stood in contrast with comments from other participants that suggested that workers should be able to provide clients with harm reduction advice based on their own professional training and knowledge (meaning that harm reduction advice was not a necessary feature of alerts). Indeed, it is well recognised that very few Australian healthcare workers undertake training specific to alcohol and other drugs, with this content largely relegated to elective courses for most professions [39–41]. Further, given the proliferation of new drug variants appearing on the drug market in Australia, as in many other countries, even workers with extensive drug knowledge can have knowledge gaps.

Some participants remarked that clients often have more knowledge about certain drugs that are in circulation than workers do. These participants challenged assumptions of the client-practitioner relationship, in which there is one-way information flow, and where it is the practitioner’s role to hold knowledge and provide education, and the client’s role to passively receive this information.

*Speaking to people who have been using heroin for longer than I’ve been alive, it’s kind of hard to educate when they have (1) already heard it, or (2) don’t care because they’ve heard the same thing over and over*. AOD practitioner, #58

As such, the dissemination of drug alert information to workers is not a straightforward information pipeline. Information is likely to be reinterpreted by workers, who draw from their knowledge of drugs, harms, and population groups in an effort to make information more relevant and useful to their target audience.

*When I think of speaking to [the] target audience, I think of targeting the different types of drug users. For instance, the audience for ‘party’ drugs (e.g. coke, MD) are often younger, less aware around drug effects and often one of the first chemical-type drugs a person will try so will be a very new experience. Compared to say heroin users who by that point have many years’ experience around drugs and often have quite a wealth of knowledge and often teach AOD workers, in my experience*. AOD practitioner, #93

In the above quote, an AOD practitioner describes factors they might consider when relaying drug alert information to a client. This quote illustrates some of the assumptions that may come into this process, namely that people who use ‘party drugs’ are likely to be inexperienced, while people who use heroin are likely to be highly experienced. While this may serve as a useful broad brush approach to tailoring information (or may be true in the experiences this practitioner), it is also important to acknowledge that this will not be true for all clients. Namely, many young people who use drugs also hold a ‘wealth of knowledge’, and people of all ages may use any substance type.

Participants described working in a variety of environments, including emergency services at festivals, outpatient therapeutic settings, hospital emergency departments, outreach in community settings, and telephone support services. Participants acknowledged that workers in each of these settings are required to provide vastly different services to clients, drawing on different kinds of knowledge and resources. As such, participants described different information needs based on their roles and work environments.

In urgent care settings where the focus is on treating and/or preventing acute harms, information about clinical presentation is likely to be more useful than information about drug chemistry (which may be more relevant to workers responsible for prescribing, dispensing or administering medicines) or preventative harm reduction (which may be more relevant to treatment, harm reduction, or peer workers who are tasked with prompting behaviour change in advance of or at the time of drug consumption).

*When patients arrive that may be drug or alcohol or affected, we actually just do a clinical assessment based on what—you know, we assess people and treat them on clinical condition, rather than looking for an image of what they may have taken. […] We will find varying substances on people and it doesn’t necessarily mean that that’s what their presenting problem is. So, we basically assess patients and not what we find on them*, Urgent care manager, #47

Notably, even within the same workplace settings, previous literature on medical alerts indicates that some workers may be more likely than others to pay attention to alerts [42]. This may be attributable to workplace guidelines, confidence, and previous training experience [42].

Participants emphasised that not all workplaces have the same level of resources and expertise available to respond to drug-related concerns. One AOD practitioner reported that hospitals vary significantly in their capacity to manage complex drug-related presentations, particularly in relation to whether or not a hospital has a dedicated team for such presentations. hese variable levels of capacity were perceived to pose a problem for workers needing to refer patients, who may have trouble identifying hospitals with the appropriate knowledge and capacity to deal with complex drug presentations.

There’s hospitals and there’s *hospitals*. There’s hospitals that have addictions teams and hospitals that don’t. So, it’s important to know […] who’s already managing it, […] is there prior experience with clinical management of this substance? Where should people go? Should they go to like [name of hospital redacted], or should there be somewhere—are they going somewhere else? What’s already known? What do we say to the hospital team? All that stuff. You can say, link to [support service name redacted], but do they know?. AOD manager, #57

These sentiments are consistent with literature indicating that local differences in expertise and capacity are a barrier to a coherent and consistent care pathway to managing adverse events in high-risk drug markets. Multiple workers with different forms of expertise may be needed to manage a presentation associated with high-risk drug use, as part of a broader system of client care [20]. However, referrers (in this case, AOD workers) may not be confident in or aware of services that are able to manage unusual drug presentations (as previously found in relation to new or novel psychoactive substances, e.g. [19]), and urgent care workers themselves may not be confident in their own ability to manage such presentations [23]. Without a system-wide approach to training and preparing the workforce with up-to-date information, drug-related presentations may be managed in a way that is inconsistent and dependent on workers seeking out information independently, rather than as part of their core training and continued professional development [20]. Importantly, previous literature has pointed to reduced workload and reduced complexity of work as being key ways to reduce alert fatigue [42].

The sheer breadth of information required to providing all possible information to all audiences would not fit into a concise alert (a key feature desired by participants, see [28]). In the workshops, there was discussion about the use of segmentation (i.e. providing select information to identified groups) to develop more targeted alerts that would provide select necessary information for identified groups. For example, participants suggested that alerts could be segmented by audience role (e.g. counsellor, peer worker, paramedic, festival promoter). However, such an approach was acknowledged by participants to require assumptions about the knowledge and experience backgrounds of the audiences, as well as their specific information needs. Participants were concerned that a segmentation approach could result in some workers missing out on important and relevant information. When asked to weigh up this tension, participants deemed that a concise alert (although strongly desired) should not come at the cost of withholding information. Participants in one workshop suggested that if segmentation was used, it would be better sorted by ‘doing’ function (e.g. I am providing harm reduction advice, I am treating acute presentations, I might use this drug), so that information is provided according to its functional relevance, rather than assumed characteristics of the individual worker and their workplace.

### TENSION 2: Minimising alert fatigue vs sharing as much information as possible

Alert fatigue (which we define as diminished capacity to pay attention to alerts) was frequently raised as a key concern for workers. Participants shared a number of factors that could contribute to alert fatigue, including workplace characteristics and alert system design. Participants expressed that it was important to avoid alert fatigue, as it could result in important messages being overlooked or deprioritised, and therefore not appropriately actioned.

*If all of a sudden this whole alert thing, everywhere’s doing it, we’re getting alerts from every which way, then it might become a bit tiresome*. AOD practitioner, #95

‘Alert fatigue’ has previously been studied in healthcare workforce settings, particularly in the context of medical alerts to assist in clinical decision-making when prescribing drugs [42, 43]. In medical alert contexts, alert fatigue has been demonstrated to significantly and strongly reduce acceptance of ‘best practice reminders’ [42]. Taking heed from these findings in other contexts, preventing alert fatigue is crucial in order for an alert system to achieve its intended purpose of informing the practice of health and community workers.

A common sentiment in workshops was that capacity to give due attention to drug alerts would be diminished by workplace demands. Participants described being ‘constantly bombarded with emails’ (regional AOD worker, #159) in busy AOD and urgent care workplaces. With many important pieces of information presented to them each day, alerts would be competing for attention with many other forms of information.

*We all get so many emails in a day, so to be able to really identify that it is an alert will be really, really important*. Regional AOD Manager, #72

One participant described this as ‘information overload’ (AOD Manager, #57). A similar concept can be found in literature relating to medical alert fatigue, where there is ‘receipt of a large quantity of information along with insufficient time or cognitive resources to distinguish relevant from irrelevant information’ [42]. Previous work has described the busy environments in which healthcare work takes place, among a ‘cacophony of warning alerts, pop-up messages, mandatory tick boxes, a Sisyphean inbox, and maddening documentation’ [44], indicating that such concerns are likely to be reflected more broadly in workplace settings beyond AOD and urgent care. Importantly, previous literature has pointed to reduced workload and reduced complexity of work as being key ways to reduce alert fatigue [42].

Another contributor to alert fatigue could be receiving alerts too frequently. As an example of their previous experiences of alert fatigue, one participant described that receiving daily government COVID-19 updates had led them to delete the emails because they had become accustomed to their content.

*It’s like those Covid ones you get every day. I just don’t bother to look at them, because I know what’s in them from there*. AOD practitioner, #112

Previous research has described this effect as ‘desensitization’, where ‘repeated exposure to alerts leads to declining responsiveness’ [42]. The experiences of our participants indicate that alert fatigue is not only of concern within a high-risk drug alert system, but would also be compounded by the receipt of multiple alerts and other forms of information.

Multiple alerts relating to the same drug event can also cause confusion and make it more difficult to recognise threats among the noise of other information. One participant noted that they had experienced confusion when receiving the same information about one substance from multiple sources. In one case, a central agency disseminated an alert, which was then reshared by other organisations, leading to confusion about whether each alert did in fact refer to the same case. Another participant described this confusion when health agencies share alerts from other jurisdictions. While acknowledging that this can be useful information due to the movement of drugs across boundaries, the participant noted that this led to the same or similar alerts being shared multiple times by different organisations.

*With the alerts in New South Wales, sometimes I wonder with some of those alerts, because you’ll get another one relatively soon and I’m thinking, could this be a similar batch? Or is it a different batch that’s come out? Then having lots of different alerts coming across from all different states. I don’t know how, if there’s any way you could make it relatively uniform in that sense, like across the board, we know that this is an alert and this is a danger in a sense*. AOD practitioner, #93

This issue of multiple alerts echoes problems identified in relation to medical alert systems, in which a significant proportion of alerts are ‘repeats’, where the same alert is provided to a healthcare worker multiple times [42]. This contributes to healthcare workers deprioritising this information, or even deprioritising information from the system as a whole [43].

Previous medical alert research has warned that new technologies and interventions designed to improve healthcare service can inadvertently contribute to broader information overload, negatively impacting the quality of work in healthcare service settings [44]. More than simply being a nuisance, ‘clinically inconsequential alerts’ [43] or ‘false alarms’ [42] (that is, alerts that are uninformative or that do not represent significant risk) contribute to alert fatigue and can increase risk of client harm through sub-optimal decision-making.

Being selective about when to releasing alerts was suggested as a way to avoid alert fatigue, with participants emphasising that the purpose of alerts was to notify audiences about high-risk situations, rather than providing general information about fluctuating drug markets. Participants suggested that alerts should be reserved for cases in which substances were sufficiently out of the ordinary, represented a sudden or dramatic change, were being missold, or in cases where harm was likely to occur. Participants noted that in illicit drug markets, consumers are likely to be accustomed to fluctuations as a normal feature of the market. As such, information about unremarkable fluctuations may not be ‘alert-worthy’.

*I think maybe also an alert that should perhaps be if there’s going to be harm associated with taking or using that substance. If it’s something that’s more just general information then maybe not an alert is appropriate. The reason I’ve just—I don’t know, if too many alerts come out people are going to get fatigued by them and just think oh well, you know, there’s just another alert about strong white powder or whatever*. AOD practitioner (non-metropolitan), #22

Conversely, deciding to *not* disseminate alerts to prevent fatigue raised questions around how best to strike a balance between minimising information overload, and gatekeeping information. With such a diversity in workforce knowledge needs and backgrounds, choosing to withhold information could prevent professionals from accessing information relevant or useful to their practice, decision-making, or interpretation of alert information. Participants also flagged that contextual information about drug alerts (e.g. information about impacts as they unfold over time) is subject to change rapidly. In such situations, releasing alert updates may risk contributing to information overload and alert fatigue, but not releasing alert updates may risk recipients not being able to access important information, at the discretion of the alert organisation.

Multiple participants suggested that a severity rating system could mitigate alert fatigue, by helping audiences to quickly assess the urgency of the alert. This could take the form of a two-tier system, or a traffic light system to demarcate different tiers of urgency.

*I like that fire alert type of system; that’s something that people are used to and I think that having the colours are good for people who—English might be their second language and that if it is in the red, even I, if I had like an SMS, I’m going, oh, I should be read that straightaway, as opposed to, oh look, it’s just, hey, for your information, and it’s a green. I can go, okay, well, I can read that later on; or I can read it tomorrow*. Urgent care manager, #42

Participants discussed benefits and drawbacks to different types of tiered systems. A two-tier system would be less complicated to categorise and interpret, whereas a traffic light system (three-tiers) would allow for more nuance. However, any rating system would come with its own complications, such as distinguishing between levels of risk, and risk level changing over time.

*I feel like a tiered system probably is useful, but at the same time it poses a risk as to, how do you tier something? How do you make someone- something high risk, moderate risk and then, oh yeah, maybe this is low risk... when actually it could become high risk?* AOD practitioner, #95

However, most participants agreed that there was merit to distinguishing the most urgent and potentially life-threatening alert cases. This difference was summed up succinctly by one participant:

*Yeah, this is not just one that’s going to make you feel bad and you might die, this is a "you’re going to die if you take it*." AOD manager, #57

Tiered alert systems have previously been used to indicate severity or level of risk in medical alert contexts and in early warning systems.Tiered alerts in medical alert systems have demonstrated greater compliance from healthcare workers who are less likely to override alerts, and more likely to take action [46]. In the drug alerts context, the Trimbos Instituut Drugs Information and Monitoring System (DIMS) in the Netherlands utilises a tiered alert system, in which a ‘Red Alert’ warning is disseminated [45]. Red Alerts include larger warning campaigns (e.g. including mass media campaigns) and are triggered only in exceptional cases ‘when serious outcomes have been reported or are expected’ [45]. Similarly, some systems delineate between ‘alerts’ and other forms of drug content information, which are still important to disseminate but not hazardous enough to warrant an alert, or which may call for different levels of action. For example, the European Monitoring Centre for Drugs and Drug Addiction (EMCDDA)’s early warning system uses four forms of risk communication that signal the ‘importance and time sensitivity of the information’: alerts, formal notification, advisory and briefing [15]. Both of these approaches (DIMS and EMCDDA) provide a balance between disseminating information about drug market variations and higher-than-usual risk, while also reserving a form of alert for critically important alerts.

Participants also proposed a number suggestions for how to design an alert system to minimise alert fatigue. Distributing all alerts through a central agency would decrease the volume of alerts and eliminate confusion relating to whether alerts disseminated by multiple organisations refer to separate issues. Participants also suggested that targeting alerts could counter alert fatigue, by ensuring that alerts are strictly relevant to that individual’s practice. One suggested mechanism was a function to self-select ‘filters’ for alerts, for example by information type (clinical management or harm reduction), or by drug type. Another suggested mechanism was to provide multiple levels of information (ranging from basic to detailed information). This would ideally reduce the amount of (non-relevant) information pushed out to alert recipients, while still allowing them to seek out further information if desired. Previous research on alerts in electronic medical records (which provide decision support tailored to patient characteristics) has demonstrated some success in filtering out alerts that are determined to have high override rates (which would indicate that many clinicians aren’t taking them seriously) [47]. An integrated alert system that is sophisticated enough to provide these kinds of tailored alerts would involve significant change to AOD service infrastructures, on par with other health service infrastructures that support healthcare workers in their decision-making (for an overview of existing clinical decision support systems, see [48]).

There are also ways that individual alerts can be designed to overcome alert fatigue. Participants shared that message formulation was also important, so that workers can quickly judge whether to read immediately or later. Alerts should also prioritise information within the alerts, focusing on harms first and providing less essential information later. Design and text can be chosen thoughtfully so that alert materials are immediately recognisable as alerts (rather than non-urgent information or documents). For example, distinctive alert designs could be used, and ‘ALERT’ can be used in email titles for quick and easy identification. Participants also suggested that having consistent design would make them distinctive and easily recognisable amongst the noise of other information.

Overall, our findings are consistent with previous literature that point to a pervasive and persisting problem of alert fatigue, which must be balanced with the need to share important and dynamic information with health and community workers to inform their decision-making. There are a number of measures that can be taken to reduce alert fatigue at the workforce level, the alert system level, and also at the alert design level.

### TENSION 3: Building trust in a prohibition world

A contextual factor that underlies these tensions is how historically radical harm reduction principles are adopted into determinedly 'apolitical' institution-led initiatives [49]. Designing drug alerts is likely to involve a range of stakeholders, including people who use drugs, government departments, law enforcement agencies, and health and community workers, who as groups and individuals may have distinct political orientations and goal conflicts, creating inherent tensions in the development process [50]. Beyond individual or organisational objectives is a broader climate of political risk and management of public perceptions, which inevitably colour how harm reduction initiatives, including drug alert systems, are made possible [51].

#### Need for credibility

Participants described that it was crucial that alerts were perceived as credible. There was significant discussion around potential ‘alert scepticism’, that is, doubt that the dangers described in alerts genuinely pose significant risk to consumers. In the context of a long history of ‘all drugs are dangerous’ rhetoric, people who use drugs and people who work with this population are accustomed to sensationalised reports of adulterated substances. ‘Dangers’ and ‘risks’ described in public health messaging may be therefore perceived by people who use drugs as exaggerated, with some level of risk understood as a normal feature of drug use, and not out of the ordinary or worthy of particular concern. Accordingly, it was suggested that sending out alerts for relatively non-urgent or low-risk situations would undermine the credibility of an alert system.

*AOD manager, #57: I mean lots of undesirable stuff happens when you use substances, right?*

*AOD practitioner, #7: Like headache, nausea, vomiting.*

*AOD manager, #57: Arsehole drug dealer, you know, wait a long time, cost more than I thought. So, it’s got to be the stuff that's like, why is this bad? What’s bad about it?*

*AOD manager, #9: It’s relevant to it being an alert rather than just, you’re not going to sleep for a night and feel really crap tomorrow. People know that and they still take it, that’s not an alert-worthy thing.*

*AOD manager, #57: That’s it, and I think it undermines the credibility of the alert system if you’re actually sending out information that’s like, “Yeah, so what?”*

This dialogue describes an experience that may be unexceptional to many people who encounter drugs: alongside the desired effects of drug consumption also comes the possibility of undesired effects. These participants called for greater discernment relating to what constitutes an ‘alert-worthy’ negative side-effects, to those which can be reasonably expected, especially in an unregulated market.

Advice provided in the alert and by people disseminating the alert could also contribute to the alert’s perceived credibility. Harm reduction advice should be relevant and sensitive to the realities of experiences of people who use drugs, even where this may be at odds with prohibition logic (which might favour abstaining entirely), or strictly health-focused sensibilities (which might favour seeking advice or treatment from a health professional). In this discussion, participants described that providing advice to call emergency services (including ambulance or police) was not always a realistic proposition, as paramedic or police intervention was not always desired. This makes alternative options for harm reduction advice important. As well as being practically useful, harm reduction advice would demonstrate that alert makers understand the audience’s needs.

Consumers were described as having extensive experience, complicating the role of the practitioner as ‘educator’ when sharing alert information. Following this, participants asserted that alert messaging should avoid being condescending or patronising, or presumptive of the relative knowledge levels of the professional and consumer. Participants described that they sometimes find themselves providing harm reduction advice to a client who has greater substance expertise than them. In these situations, not knowing about particular substances undermined the worker’s credibility to a client.

*If someone comes to me and says, I’m using this, this and this, and I have no idea what that means, I look like an idiot. They think that drug and alcohol services don’t work. I’ve had someone talk about 1,4-B, which is basically GHB. I didn’t know that. I had to Google it. I had to—and I sounded like a bit of a silly boy, so I definitely would love to know about new stuff. I like knowing—the only reason I know anything about drugs is because clients tell me, "oh, you know the price of heroin has gone up because of COVID." I’m like oh, good to know. I should know that. Things like that. But yeah, it would be nice to get updates on—like even if someone says, "I’m using ice," and I’m like, "Well actually, can you just, next time you do that, this is happening at the moment, just take extra—a bit more caution when you do use it next time." Things like that*. AOD practitioner, 58

This quote illustrates that knowledge of more commonly used drugs is not always enough to be fully across how drugs are currently being consumed. However, with approximately 500 novel psychoactive substances identified around the world in 2019 [52]), it would be impossible to expect all workers to be aware of all drugs on the global market. Alongside greater specific drug training (e.g. through more standardised approaches to including drugs in healthcare curriculums), drug alerts can provide up-to-date and relevant information about new substances in local drug markets, thus preparing health and community workers to respond to substances that are less commonly used, or otherwise unlikely to be addressed in a limited AOD curriculum as part of their standard training.

#### Building trust through branding and messaging

Branding and messaging are important factors in perceived alert credibility. Institutional branding of an alert should be strategically considered. On one hand, alerts that carry institutional branding can symbiotically borrow the institution’s credibility, while also helping to build trust in that institution. However, if audiences do not think that it is appropriate for alerts to come from that institution, such branding could cause opposition to the alerts.

Participants suggested that branding should be from a health perspective. This suggestion arose when discussing an existing alert disseminated by a police forensics department. Participants described that this ‘forensic’ branding would likely hold connotations of the justice system. This would undermine trust and credibility among people who do not trust or ‘respect’ police and justice systems.

*Certainly, if it was going out to consumers probably trying to brand it a bit more from a health perspective rather than forensic in particular as a word. Because a lot of our clients would associate the word forensic with the legal system*. Regional AOD manager, #138

Previous research documents mistrust of police (and other forms of authority) from people who use drugs, based on witnessing or experiencing negative encounters with police [53]. Viewed as a structural issue, policing approaches that use a more collaborative and compassionate approach to addressing drug-related issues pose an opportunity to reduce drug-related harms in collaboration with affected communities [54, 55].

#### Credible information sources

Transparency relating to the source of information for alerts is an important part of building trust in alerts. Participants asserted that people want to know where information is from, especially in the context of alert scepticism and historically sensationalised reports of dangerous adulterants. Providing this information is an opportunity to demonstrate that alerts are reliable and verifiable.

*Was it police? Was it a hospital admission? Was it consumers reporting things? Would just be helpful for people to trust where the information is coming from. Because even if it came from the department, people go, "Well, how do you know this?"* AOD practitioner, #46

Participants also noted that different information sources would have different perceived reliability and validity, and different benefits and limitations. Providing caveats or notes would help audiences to interpret the alerts and use them appropriately. Drug checking was considered to be the most accurate, reliable and fastest way to disseminate drug alert information, especially at events, where drug checking can happen on-site, with the added benefit of proactively identifying risks (i.e. able to test and disseminate information prior to harms occurring), when compared to overdose or hospital data. Police seizures were also perceived as reliable sources of information, albeit with limited utility due to practical limitations, such as delays after going ‘through the system’ (AOD manager, #25).

Participants discussed the information that was currently available to them, in the absence of an established alert system. Some participants discussed that informal reports from hospitals were available, where hospitals shared information about presentations with AOD services (for example, unusual presentations or overdoses). This information provided useful signals to AOD services who could then warn consumers. However, while hospitals can report on what is presented to them, they are unable to provide or confirm more detailed information about substances (e.g. their contents).

*We have two sources. One from A&E [accident and emergency departments] that’s not reliable but it is information […]—not reliable in the fact that they can’t tell us what’s in the substance, all they can tell us is this is happening. […] The other one that we pass on is client information, of course around ODs but also around other substances that may or may not be what they’re supposed to be. Given that we live in [region name redacted], it’s a fairly small area. Geographically, a lot of the users that use here, don’t tend to go out of [region name redacted] or [region name redacted] area. They stay within those confines. So, a lot of the information we get from clients that we pass on, sometimes we find is very valuable yet other times, it may not be as valuable*. AOD practitioner, #65

Consumer reports were described as a main source of information for health and community workers about drugs currently in circulation. These reports include anecdotal information about experiences with drugs or drug markets. One participant even described workers as relying on consumer reports to inform their work and to forewarn other clients:

*The source of our information and early warning systems comes from our clients. They’re the best people to tell us but also, at times, it’s not always easy to verify and challenging because we’re just going on personal accounts because they don’t have testing available for them either. So, it’s just a ‘hey, I tried this and it didn’t—it wasn’t the usual and I’m worried about this’. Or, ‘I know such-and-such and they had an overdose’. Therefore it’s not—you can’t verify it enough to share it amongst the sector, either*. AOD manager, #25

A significant limitation of this information is that reports can’t be ‘verified’, in the sense that experiences may be specific to the individual, and further information about the substances (e.g. their contents) cannot be investigated.

*I think if it gets to the point where you’re looking at doing consumer feedback to inform some alerts there really needs to be that robust framework around validating it and making sure it’s worthwhile sending out. Because sometimes there’s a little bit of an impetus to an anecdote becomes a fact and there’s lots of variables that can go into anecdotal feedback from clients. So there definitely needs to be some level of validation around that whether it’s the number of clients that might report it or some sort of other mechanism to validate it a little bit*. AOD manager, #67

However, consumer reports are unique in being able to identify what is happening ‘on the ground’, providing timely information about substances of concern to consumers in-the-moment. Another consideration is that such ‘unverifiable’ consumer anecdotes may become more reliable sources of information when aggregated across multiple reports. As an example of the value of consumer experience reports, feedback systems are an important function of darknet marketplaces that sell drugs, as a way of building trust and credibility [56]. While one single report may not make a trustworthy account of risk, multiple reports are an important insight into how drugs are experienced. Therefore, despite not being ‘validated’ sources of information, they are valuable in providing information and context.

Participants suggested that these various sources of information could work together to provide higher quality information about substances. Crucially, combining these sources of information can increase drug alert credibility and usefulness. For example, drug composition analysis could be used to verify client reports and hospital presentations, and client reports could be used to contextualise drug checking, police seizures, and hospital presentations and provide evidence that they are genuinely affecting consumers.

*I can see a lot of consumers and actually a lot of workers honestly reading through it and being like, oh okay. There’s risk associated with every drug. […] But if there are consumers going out there, then like no this is actually concerning or this is what we’ve experienced with my mates and whatever, I think it validates it a little bit more. It’s less of that, don’t take any of these drugs you’re buying on the street because they’re all going to be dangerous kind of rhetoric*. AOD practitioner, #61

While participants ideally wanted information that is demonstratable as being trustworthy and credible, information that has potential to reduce risks or harms should not be withheld. In lieu of being able to corroborate reports with drug analysis data, some services have developed their own validation mechanisms. For example, some teams discuss reports in team meetings, and decide to pass on information to other clients if multiple clients have reported the same thing. One participant described an informal workplace process for ‘verifying’ hospital or consumer reports, where, in cases where there is information that could prevent or reduce harm but is ‘unverified’, there is a preference to pass on that information rather than withhold it, especially if the potential dangers were considered high.

*At [hospital name redacted], we definitely pass information on, even though we can’t verify it, but we sort of assume it’s better to pass information on to our clients rather than not, given particularly if we’re dealing with high-grade heroin or bad batches of ice that have put people in hospital*. AOD practitioner, #65

As an additional verification system, participants suggested that drug alerts could be accompanied by a feedback system. For example, people could provide ratings and feedback to demonstrate how and whether other people have used and passed on the information to clients. Knowing how other health and community workers had used this information would give other workers confidence to share it with their own clients.

### TENSION 4: Avoiding unintended alert consequences

This theme describes the complex task of avoiding unintended consequences that may arise when disseminating an alert. What the ‘intended consequences’ are may vary between people (for example, reduction in harm vs reduction in use) (see 28 for a description and discussion of participants’ desired impacts). We use ‘unintended consequences’ here to refer to adverse outcomes for people who use drugs, including behaviours enacted by people who use drugs, as well as other people.

Participants described that messaging can inadvertently *promote* substances, rather than act as a deterrent. Participants expressed concern that there was potential for drug alerts to have the opposite effect to what was intended, especially messaging about ‘strong’ substances being interpreted as desirable and therefore encouraging people to seek that particular substance. One participant noted that they themselves were hesitant to pass on some alerts to clients, due to their concerns that the wording would encourage their client to seek out that substance.

*My local alerts come through [redacted organisation] or through the health department. I always worry, I want to send it out to my clients, but […] I haven’t used a drug in 20 years, I look at it and I go oh my god the heroin looks great. There’s something in it that’s like—because the language they use, the way they do it, like warning, warning, really strong heroin on the streets. So, that’s what stops me from passing it onto clients now, but I think it would be good to have something that we could pass onto clients if necessary*. AOD manager, #57

There is also a need to manage scrutinous public responses to drug alerts as a potential unintended adverse consequence. A key tension identified here was that for an alert to prevent as much harm as possible, it must be widely distributed; however, wide distribution likely meant the attention of ‘anti-drug public’ (including friends and family) who may form negative opinions about the alerts, or people affected by alerts. As a first step, alert creators must be careful to craft alerts that do not use stigmatising language, to avoid inadvertently providing fuel to stigmatisation.

*I’ve been around a long time and I have seen how the media love to jump on this stuff and talk about, “Where’s my hand-delivered chardonnay? Where’s the chardonnay van? You’ve got a syringe van, your injecting centre. Where’s my chardonnay drinking centre?” […] Essentially, in order to be useful it has to be widely distributed and really public. There’s going to be a lot of media attention on it, […] worried parents and loved ones looking at it and […] getting in a flap about it. I think in order for it to have a lifespan, a long lifespan, some of those things need to be taken into account*. AOD manager, #57

This point also brings into focus the importance of broader stigma-reduction initiatives, to target things that aggravate anti-drug sentiment. This includes initiatives such as those that target stigmatising language (e.g. ‘The Power of Words’, [39]) and those that call out stigmatising representations of drugs and people who use drugs in the media [57]. Rather than an ancillary concern, stigma poses a significant challenge to implementing harm reduction initiatives such as drug alerts, particularly because of the influence of public support on service funding and provision, and impacts on access [58, 59].

Participants also expressed concern about the potential for drug alerts to be used to justify more punitive responses to drugs. Potential scenarios raised by participants included concerns that sharing on social media could lead to being noticed by authorities, or that event organisers could be held liable for harms or deaths if given ‘pre-warning’ of risks with alerts. Participants were also concerned that the dissemination of drug alerts could be used to justify greater policing.

*Well, I have concerns that if you put out an alert, a public alert, that the police will actually come to fix that problem. And given that we don’t live in a harm reduction world, we live in a prohibition world, their way that they’ll fix that is by heavy—heavily policing that area to stop that activity or drug being sold*. AOD practitioner, #65

Participants also conceded that, as part of the nature of drugs in the context of prohibition, mitigating all risks would be impossible. As one participant remarked (and as is the case with many public health initiatives), there is always a risk of unintended consequences.

*I think there’s always going to be a risk of oh that’s a really strong pill, and you see it on some of the alerts in the UK where people are like oi, come on John, let’s go and get these. Whereas there’s still going to be a whole load of other people that are actually taking that alert seriously*. AOD manager, #9

In summary, participants stressed the importance of  carefully and thoughtfully crafting alerts to minimise unintended consequences. They also noted that undesirable 'unintended' consequences were made more likely by stigma, variation in individual behaviour, prohibition logic, and lack of information about drug markets.

### TENSION 5: Designing alerts for workers to reduce harms, when consumers are the affected population

Our co-design process was formed to design alerts for health and community workers, arising from an opportunity to utilise data from a restricted data source. However, during the workshops it became apparent that it was not possible to design alerts without considering the needs of consumers, as the population ultimately affected by drug alert information. Participants explained that getting alert information to consumers was their priority, and that alerts should be designed with this end-goal in mind.

Participants were adamant that workers should not be ‘gatekeepers’ of important drug alert information. A recurring sentiment was thatworkers should not have any more information than consumers. Given that the purpose of drug alerts is to reduce harms to people who use drugs, participants did not believe that health and community workers should solely manage alert dissemination. Additionally, for those workers whose roles involved responding to acute presentations, they noted that they would prefer to prevent presentations in the first place by providing consumers with this information.

*It would be really nice to get an alert sent to the community, to the people at the front face, rather than us having an alert as clinicians and managing the after-effects of what’s happened. […] we’re in a crisis when we come to emergency, so I think reach the people where it matters and where it really affects*. Urgent care manager, #47

To effectively disseminate information to people who use drugs, peer networks would need to be utilised. Participants noted that designing alerts only for the health and community workforce would limit their reach, especially as a large proportion of people who use drugs do not access services. Therefore, participants said that it was important to actively target people who use drugs who are *not* accessing services, rather than developing alerts that are *only* designed to reach people who engage with services.

*Our clients tend to talk—being a small town, they tend to talk to each other more so than us. I think we—here, I would like a drug alert to be able to go out to people that aren’t engaged with us who are still using. That’s the cohort that I think we really need to try and target. Other people that are actively using and not seeking help from services*. Regional AOD practitioner, #133

AOD workers (including and especially peer networks) can champion dissemination and ‘push’ alerts to clients, who may not otherwise see or seek out alerts. Designing alerts for urgent care workers is another key opportunity for alert dissemination for people who use drugs who are not typically engaged with AOD services.

*I would say we still do a huge amount of harm reduction advice in the emergency department, because we see a lot of patients who aren’t at that time engaging with other AOD services, or specialist services. So, the emergency department might be the only place where they are getting that face-to-face harm reduction advice* AOD practitioner & manager, #96

It is important to involve consumers in the design process, regardless of whether the alert is targeted towards workers or consumers. Participants speculated that consumers may want different information from an alert, compared with, and discussed methods for dissemination and designing alerts targeted towards consumers. However, if making consumer alerts, it would be crucial to involve consumers in their design and development, and not just at later stages as a vessel for dissemination. Having peer input would help to analyse messages to minimise unintended alert effects, such as inadvertently promoting the drug (as discussed in Tension 4).

*It’s also on the awareness of the peer we’re working with. Like if I told some of my clients hey, it’s really powerful heroin going around in [inner-city suburb] at the moment, […] they’d all flock there to go and score some, so it’s actually about what message we’re trying to get across. Whereas it would make some more aware, “Okay, I’ll be really careful, I’ll let my mates know and all that sort of stuff.” […] Having some good peer input would be important […] because they will actually help you to analyse the message you’re doing*. AOD practitioner, #112

Ultimately, given that the purpose of drug alerts is to reduce harms among people who use drugs, the needs of consumers cannot be ignored in the alert design process. Previous research has demonstrated that meaningfully engaging with people who use drugs in the design, provision and evaluation of harm reduction services is beneficial to both the empowerment of people who use drugs, but also improved outcomes of services through ‘fostering communication, building trust, increasing knowledge, and reducing stigma and discrimination to remove barriers and increase utilization of harm reduction services’ [60] (see also [61–63]).

## Recommendations and implications

Below, we propose some suggestions to navigate these tensions when designing drug alerts and drug alert systems. We provide these with the caveat that both tensions and solutions depend on the context, target audience and setting of alerts, and should be considered accordingly.

### Support organisations and workplaces to tailor, disseminate, and respond to alerts

At a broader system level, changes to build health and community workforce capacities to respond to high-risk substances is needed. This includes integrating drug/drug use modules into health training curriculums and upskilling workers to increase harm reduction literacy. Organisations could also be supported to tailor and disseminate alerts with supporting resources on best-practice information dissemination information, and suggestions for how and when to disseminate information.

While having a central organisation as a source of alerts helps to reduce confusion, this central organisation should focus on developing centralised, top-level alerts, acting as a comprehensive (but easy-to-understand) ‘source of truth’, in which other organisations can repackage alert information in a way that is tailored to their audiences. Workplaces may be better placed than central alert organisations to tailor messaging to their staff or clientele. Partnering with workplaces and local organisations is an opportunity to disseminate more nuanced information that is responsive to local contexts.

This work could include building capacity for appropriately resourced ‘alert champions’ (e.g. employees with high knowledge of or interest in drug alerts or harm reduction contents) who can identify key messages that are relevant to the nuance of their workplace, tailor messages to their workplace and disseminate alerts among their workplace. Nominated alert champions within organisations and across networks may be useful for establishing credibility of new alert systems, minimising alert fatigue and more effective, ongoing information-sharing settings. The use of champions has been reported to contribute to intervention success via promoting its value and sustaining interest and engagement [64]. A challenge of this ‘workplace champion’ approach would be ensuring sustainability when the champion moves workplaces or roles. This could be ameliorated, by example, by including a specific position description for this role.

### Specifically build dissemination strategies into the alert system

Currently in Australia, alert ‘translation’ is often led by peer-led harm reduction organisations who develop consumer-focused versions of alerts (for example, Harm Reduction Victoria and NSW User and AIDS Association, see supplementary file in [28] ). This is a pragmatic approach to tailoring alerts to diverse audiences, making the most of the expertise of local services who are familiar with their client characteristics, allowing for tailored messaging approaches at scale. However, this can be time and labour-intensive for other organisations, and requires investment to ensure quality, so that the credibility of alert systems is not undermined by inconsistent messaging and perceived credibility. As such, resourcing these activities as part of an alert dissemination strategy would be important to ensure that tailored messaging is consistent with the broader alert system and would allow for micro-level feedback to organically feed into the broader alert system.

When disseminating alerts to services, a ‘media kit’ that includes multiple formats for those services to review and disseminate as appropriate would ensure that services can provide relevant messaging to their audiences. A media kit could including information about communicating harm reduction messaging sensitively (e.g. including key takeaways on stigmatising language from [39]).

### Provide transparency about the alerts and alert process

Alert system credibility and trust is achieved organically over time, by providing consistent and high-quality information. An alert system would benefit from being transparent about who runs the alert system, for what purpose, and how it is funded (e.g. DIMS in the Netherlands provides transparent information about their processes, [65]). Transparency around decision-making processes (e.g. how does the alert system decide whether or not an alert is released?) could also bolster faith that alerts are important and chosen for health- and harm reduction-related reasons, rather than political or anti-drug messaging reasons. This has been explored in recent public health literature that explores the importance of trust and transparency in vaccine rollouts [66, 67]. Where possible, providing information about the source of alerts (e.g. hospital, drug checking service, police data, peer reports) would build trust in the authenticity of alerts. Transparency relating to the limitations of the data, system, or claims of alerts could also built trust. In some cases, information may be incomplete, unclear, or unable to be communicated to protect patient privacy. In these cases, rather than entirely removing any mention of this information, greater transparency could be promoted by redacting information or explaining why the information is not included in an alert.

### Involve people who use drugs in the design of alerts

As the scope of our project was to design alerts for healthcare professionals, our discussions were framed in a way that assumed that healthcare professionals were alert ‘end-users’. However, our conversations fundamentally revolved around how drug alerts might impact people who use drugs, as the most affected population. People who use drugs should ideally be involved in the initial design process, but also involved in ongoing dialogue. This is because people who use drugs understand their drug-using contexts best, and can provide crucial, up-to-date insights that can help to avoid unintended consequences. Previous research has noted that people who use drugs may view alerts with skepticism or may not consider drug information to be credible. Similarly, drug-related information with a focus on providing action advice that focuses on abstinence or prevention of uptake is unlikely to resonate with or be useful to people who currently use drugs, and their drug-using contexts. This also presents a powerful opportunity for meaningful dissemination, among so-called hidden populations of people who use drugs. While disseminating drug alerts to health and community workers provides breadth across the population, dissemination via peer/social networks provides opportunities for depth of message dissemination.

### Build feedback mechanisms into the alert system

Drug alerts and drug alert systems should be responsive to dynamic drug markets and drug-using contexts. Given the many stakeholders involved in such a system, it is important that implementation is supported by ongoing evaluation that considers different audiences, goals, and settings.

Evaluation can relate to a number of outcomes. From a workforce perspective, it would be useful to monitor how they are integrated into practice. Monitoring alert fatigue would also be useful to maximise impact, and could involve measuring how often an alert is ignored or actioned [68]. Evaluation should also monitor consumer-level impacts of drug alerts, such as desired behaviour change, or unintended consequences. Evaluation should ideally be conducted both in the form of formal and comprehensive program evaluations [10, 11, 16, 17, 25], and also smaller-scale ongoing evaluations that inform continuous improvements to alert systems through built-in feedback mechanisms.

Feedback mechanisms are currently built-in to other alert systems, such as New Zealand’s High Alert website which provides a mechanism for people to report experiences of drugs highlighted by alerts [69]. Alert system feedback can also be automated, taking signals from health and community worker behaviour (such as whether the alert is digitally actioned or overridden) [47]. Regardless of data collection method, trialling and evaluating different ways of producing alerts on a case-by-case basis would be an important part of ongoing development and evaluation to establish best practice across multiple contexts.

### Use risk-assessment and decision-making processes to develop tiered alerts

Differentiating between higher- and lower-risk alerts can help to mitigate alert fatigue. However, binary outcomes of alert/no alert in decision-making frameworks lead to difficult decisions to not release alerts, thus potentially withholding important, useful, or desired information. Ideally, an alert system should transparently provide information available about drug contents, so that audiences can access information that is useful or relevant to them, while also not overwhelming audiences with a continuous flow of frequent alerts. It is also possible to create dynamic tiers, where an alert’s severity or urgency can be increased or decreased [70]. There are many examples of how this is done in practice, including Trimbos Instituut’s Red Alert and the EMCDDA’ s early warning system (as described in Tension 2). Established alert system protocols also provide examples for how decision-making frameworks to triage high-risk drug information [15]. Future research could investigate what contextual factors important for practical decision-making in relation to drug alerts (as previously explored in the medical alert context, [68]).

### Limitations

Our participants comprised a self-selected sample of health and community workers, who are therefore likely to be more engaged or interested in drug alerts or harm reduction than other workers. Uptake and engagement with an alert system may be qualitatively different among people who have lower interest in these topics, particularly in relation to alert fatigue where drug alerts may be a significantly lower priority than other forms of information. Feedback and evaluation occurring in a drug alert trial may provide greater insight into more general workforce reception and perceptions of drug alerts. We specifically recruited workers in AOD and urgent care settings, as two critical healthcare sites accessed by people who use drugs, for drug-related problems. While we have generalised our findings as indicative of broader healthcare workforce needs, there are likely to be other contextual factors that lead to different experiences and needs of other healthcare workforces, included people working in pharmacy or general practice settings.

## Conclusion

We identified a number of tensions in the process of co-designing alerts for health and community workers, that were specific to the audience (a diverse workforce), nuances of the subject matter (a range of substances, and consumption practices relating to them), regulatory environment (in which drugs and drug use are criminalised), and political environment (in which the voices of people who use drugs are often side-lined, while being subjected to stigma), in which we were designing the alerts. We considered these tensions in the design of our final drug alert templates, which can be found in our other paper [28]. While the tensions remain, we worked with participants to identify how we could design alerts that seek a balance between the tensions. Overall, we found that there was certainly no ‘one-size-fits-all’ solution. Rather, these findings highlight the need to be aware of competing priorities in the design process. These findings have implications for people creating drug alerts and other drug-related health risk communications. Overall, we identified five interconnected tensions that should be considered in the design of drug alerts:

1. There is a need to provide comprehensive information to meet the information needs of a diverse group of professionals with varying knowledge levels. However, alert messaging must be clear and concise, and relevant to the work of individuals.
2. Professionals in the AOD space experience ‘information overload’, which may lead to ‘alert fatigue’. However, it’s important that we do not gatekeep information, and that information should be available to those who want it.
3. Alert design and dissemination must be perceived as credible, to avoid ‘alert skepticism’. But ‘alert skepticism’ is a product of a broader context of criminalisation, stigmatisation, and sensationalism.
4. Alerts must be carefully designed to achieve ‘intended effects’ and avoid unintended effects. It is impossible to control all potential effects, and desired outcomes may vary between actors.
5. The scope of the project was to develop alerts for professionals to use in clinical settings. However, people who use drugs are the end-user and must be kept front of mind in the design process.

This research has implications not only for people creating drug alerts, but also anyone developing drug-related health risk communications. It is inevitable that there will be tensions and complexities in the design of high-risk drug alerts. Paying attention to these tensions enables us to understand the nuance of how people might interact with the alerts in practice, and to pre-emptively consider how that might impact implementation. Our research highlights the need to understand context in the design process, so that these complexities can be both surfaced and addressed to minimise harms and maximise relevance and uptake of alerts. Acknowledging that it is unlikely that any single alert design will ever be a one-size-fits-all solution, considering the broader context also facilitates the identification of opportunities to draw on broader infrastructures to facilitate positive outcomes.

## Supplementary Information

**Additional file 1.** Coding Frame.

## Data Availability

Qualitative data generated and/or analysed during the current study are not publicly available to protect the privacy of research participants who attended digital workshops. Deidentified data from the online scoping survey may be made available from the corresponding author upon reasonable request.

## Abbreviations

AOD: Alcohol and other drugs
EMCDDA: European monitoring centre for drugs and drug addiction
