# Correction: ‘We don’t live in a harm reduction world, we live in a prohibition world’: tensions arising in the design of drug alerts

**DOI:** 10.1186/s12954-023-00784-z

**Published:** 2023-04-25

**Authors:** Isabelle Volpe, Rita Brien, Jasmin Grigg, Stephanie Tzanetis, Sione Crawford, Tom Lyons, Nicole Lee, Ginny McKinnon, Caitlin Hughes, Alan Eade, Monica J. Barratt

**Affiliations:** 1 Social and Global Studies Centre, RMIT University, Melbourne, Australia; 2 Drug Policy Modelling Program, Social Policy Research Centre, UNSW Sydney, Sydney, Australia; 3 Turning Point, Eastern Health Statewide Services, Richmond, Australia; 4 Monash Addiction Research Centre, Eastern Health Clinical School, Monash University, Melbourne, Australia; 5 Harm Reduction Victoria (DanceWize), North Melbourne, Australia; 6 Department of Health, Victoria State Government, Melbourne, Australia; 7 360Edge, Melbourne, Australia; 8 National Drug Research Institute, Curtin University, Perth, Australia; 9 Law and Commerce, Flinders University, Adelaide, Australia; 10 National Drug and Alcohol Research Centre, UNSW, Sydney, Australia; 11 Safer Care Victoria, Melbourne, Australia; 12 Department of Paramedicine, Monash University, Melbourne, Australia; 13 Digital Ethnography Research Centre, RMIT University, Melbourne, Australia

**Correction:  Harm Reduction Journal (2023) 20:3** https://doi.org/10.1186/s12954-022-00716-3

Following publication of the original article [1], the affiliation for the author Stephanie Tzanetis has been updated as “CanTEST (Direction Health Services, Pill Testing Australia, Canberra Alliance for Harm Minimisation and Advocacy), Canberra, Australia.”

The original article has been corrected.
