# Supplementary material for: ‘We don’t live in a harm reduction world, we live in a prohibition world’: tensions arising in the design of drug alerts
Source: Harm Reduct J. 2023 Jan 9;20:3. doi: 10.1186/s12954-022-00716-3 (PMC9829230; doi:10.1186/s12954-022-00716-3)
Supplement: Supplementary file 1 — Additional file 1. Coding Frame. [file 12954_2022_716_MOESM1_ESM.docx]

**Code Frame: RapidDAV**

1. Objectives/goals
   1. Reduce drug-related adverse events
   2. Individual behaviour change (e.g., reduced use)
   3. Improved professional response (e.g. clinical)
   4. Education (information/knowledge exchange)
   5. Other objectives
2. Deciding to issue an alert
   1. Evidence of harm
   2. Level of risk
   3. New or unexpected substances
   4. Relevance to stakeholders
   5. Timing and recency
   6. Other decisions
3. The Alert
   1. Content (e.g. language, information, clarity)

3.1a Substance information

3.1b Context of the sample

3.1c Language considerations

3.1d Practical advice for PWUD

3.1e Practical advice for clinicians

3.1f Further information

- 1. Framing (e.g. tone, focus, narrative)
  2. Design (e.g. visual)
  3. Other alert

1. Dissemination
   1. Alert format (e.g. email, website, poster, SMS)
   2. Distribution from source
   3. Circulate in professional networks
   4. Sharing with public (e.g PWUD)
   5. Other dissemination
2. Sources
   1. Alert source
   2. Drug information source
   3. Other source
3. Audience
   1. Professional (PWW-PWUD)
   2. PWUD
   3. Public
   4. Other audience
4. Challenges
   1. Alert fatigue
   2. Knowledge levels
   3. Credibility (e.g. alert source, drug information source)
   4. Other challenges
5. Opportunities
6. Anything else/misc
7. Good quotes
